# Artificial Intelligence in Medical Assessment: Reliability and Performance of Multimodal Large Language Models in a High-Stakes Licensing Examination

**DOI:** 10.3390/bs16050822

**Published:** 2026-05-19

**Authors:** Ibrahim Güler, Gerrit Grieb, Armin Kraus, Philipp Moog, Uzay Cambaz, Ezgi Yavasca, Henrik Stelling

**Affiliations:** 1 Department of Plastic, Aesthetic and Hand Surgery, Otto-von-Guericke University, 39120 Magdeburg, Germany; armin.kraus@med.ovgu.de; 2 Department of Plastic Surgery and Hand Surgery, Gemeinschaftskrankenhaus Havelhöhe, Kladower Damm 221, 14089 Berlin, Germany; gerritgrieb@gmx.de; 3 Department of Health Management, Friedrich-Alexander-Universität Erlangen-Nürnberg (FAU), Lange Gasse 20, 90403 Nürnberg, Germany; henrikstelling@googlemail.com; 4 Department of Plastic Surgery and Hand Surgery, RWTH Aachen University, Pauwelsstrasse 30, 52074 Aachen, Germany; 5 Department of Plastic Surgery and Hand Surgery, Klinikum Rechts der Isar, Technical University of Munich, Ismaninger Str. 22, 81675 Munich, Germany; philipp.moog1@mri.tum.de; 6 Faculty of Medicine, Eberhard Karls University of Tübingen, Geschwister-Scholl-Platz, 72074 Tübingen, Germany; uzay.cambaz@student.uni-tuebingen.de; 7 Department of Nuclear Medicine, Klinikum Ernst von Bergmann, 14467 Potsdam, Germany; ezgi.yavasca@charite.de; 8 Practices for Nuclear Medicine, Rubensstraße 125, 12157 Berlin, Germany

**Keywords:** artificial intelligence in assessment, medical assessment, large language models, multimodal AI, medical licensing examination, Turkish Medical Specialization Entrance Examination (TUS), psychometrics, reliability analysis, reproducibility, AI benchmarking

## Abstract

Artificial intelligence (AI) is increasingly integrated into assessment contexts, yet evidence on the reliability and measurement properties of large language models (LLMs) in high-stakes evaluation settings remains limited. This study examines the performance and reproducibility of contemporary multimodal LLMs in a structured medical assessment environment. A cross-sectional dual-setup design was applied using a complete national medical licensing examination (240 multiple-choice items, including image-based questions). Setup 1 evaluated ten models in a single run to characterize overall performance. Setup 2 assessed six models across five independent runs each to quantify measurement stability. Accuracy with 95% confidence intervals, inter-run agreement using Cohen’s kappa, and paired comparisons using McNemar’s test were analyzed. Accuracy ranged from 72.08% to 92.92%. All models demonstrated near-perfect inter-run agreement (mean κ ≥ 0.96) with minimal variability. After correction, only a small number of pairwise comparisons remained significant, indicating convergence among leading systems. In an exploratory submodule, performance on the small set of image-based items was comparable to or slightly higher than performance on text-only items. These findings demonstrate that multimodal LLMs achieve high accuracy and high inter-run reproducibility on a large-scale assessment, supporting their use as objects of AI-based assessment research while leaving questions of cognitive equivalence with human examinees beyond the scope of accuracy-based evaluation.

## 1. Introduction

Large language models (LLMs), and their multimodal extensions (MLLMs), have introduced new possibilities for the design, evaluation, and study of assessment instruments across educational and clinical settings. Built on transformer-based architectures (Vaswani et al., 2017), these systems process complex test items, integrate multimodal information, and produce responses that can be evaluated against scoring rubrics or answer keys. Their growing deployment in assessment contexts raises fundamental questions about measurement quality, including the accuracy, consistency, and generalizability of AI-generated responses under standardized testing conditions (Owan et al., 2023; Bulut et al., 2024).

Medical licensing examinations represent a particularly informative domain for studying these properties. As high-stakes assessments designed to assess clinical reasoning, knowledge integration, and diagnostic judgment in human examinees, they offer psychometrically rigorous item sets with well-defined scoring criteria and established performance benchmarks. Several studies have reported that LLMs achieve scores at or above passing thresholds on the United States Medical Licensing Examination (USMLE), with some systems exceeding 80 to 90 percent accuracy (Kung et al., 2023; Knoedler et al., 2024; Nori et al., 2023; Roos et al., 2023; Mihalache et al., 2023). These findings have stimulated interest in AI as both a subject and a tool of assessment research, with potential applications in automated scoring, adaptive testing, and item analysis (Li et al., 2025; Mendonça et al., 2025; Thomas et al., 2026; Yan et al., 2023).

Despite this progress, the evidence base presents several gaps relevant to assessment science. Most existing evaluations are concentrated on English-language examinations, predominantly the USMLE (Kung et al., 2023; Knoedler et al., 2024; Nori et al., 2023; Roos et al., 2023; Mihalache et al., 2023; Mihalache et al., 2024; Penny et al., 2024; Gilson et al., 2023; Abbas et al., 2024; Bicknell et al., 2024; Garabet et al., 2023; Balu et al., 2025; Yang & Chen, 2025). Studies conducted on non-English assessments typically involve partial item sets, limited multimodal coverage, or single-run evaluations that do not quantify measurement stability (Sismanoglu & Capan, 2025; Pamuk et al., 2025; Meyer et al., 2024; Kipp, 2024; Şensoy & Çıtırık, 2025; Jung et al., 2023; Sabaner & Yozgat, 2025). Even when full examination sections have been studied, image-based items are frequently omitted owing to inconsistent multimodal capability across model generations (Jung et al., 2023). As a result, robust, full-assessment multimodal benchmarks outside the English-speaking context remain scarce, and reproducibility data are largely absent from the literature.

From the perspective of assessment science, the absence of reliability evidence represents a critical limitation. Measurement theory requires that any assessment tool demonstrates consistent performance across repeated administrations (Cohen, 1960; Landis & Koch, 1977). Without such evidence, accuracy estimates may reflect stochastic variation rather than stable measurement properties. Yet the majority of published LLM evaluations report only single-run accuracy without quantifying inter-run agreement, confidence intervals, or within-system variability.

Within classical test theory, reliability refers to the reproducibility of a measurement across repeated administrations, while construct validity concerns whether the obtained scores reflect the cognitive ability that the instrument is intended to capture. In an AI-based assessment paradigm, both must be examined separately. A response process that produces highly consistent answers across runs may still rest on mechanisms unrelated to the underlying construct, and high reproducibility therefore cannot be interpreted as construct equivalence with human examinees. Accordingly, the present design addresses the reliability of LLM outputs but does not evaluate construct validity with respect to human clinical reasoning. Anchoring the present evaluation in this framework reframes the question from ‘Can the model pass?’ to ‘Can LLM outputs be characterized using established psychometric methods, independent of the underlying response process?’

The Turkish Medical Specialization Entrance Examination (Tıpta Uzmanlık Eğitimi Giriş Sınavı, TUS) provides a suitable framework for addressing these gaps. The TUS is a high-stakes examination administered by the Turkish Assessment, Selection and Placement Center (Ölçme, Seçme ve Yerleştirme Merkezi, ÖSYM), consisting of 240 multiple-choice items spanning the full domain range of basic and clinical medical sciences (ÖSYM, n.d.; Aydoğan & Keskin, 2022). Its combination of non-English administration in an agglutinative language (Turkish), broad disciplinary scope, and inclusion of image-based items offers a challenging assessment context for studying LLM measurement properties. Existing TUS-related LLM evaluations offer valuable initial insights but are limited by partial item coverage, absence of multimodal analysis, or insufficient methodological rigor with respect to multi-run reliability and statistical comparison across systems (Sabaner & Yozgat, 2025; Gencer & Gencer, 2024; Aygül et al., 2024; Kılıç, 2023).

Several structural limitations extend beyond the TUS literature. Prior non-English benchmarks often pool items from different examination cycles, reducing ecological validity, and rarely incorporate established reliability metrics such as Cohen’s kappa or paired significance testing using McNemar’s method (Cohen, 1960; Landis & Koch, 1977; McNemar, 1947; Sedgwick, 2014; Armstrong, 2014; Nakagawa, 2004). Methodological transparency also remains inconsistent, with many studies failing to document prompting strategies, interface conditions, or model identifiers. These omissions reduce comparability and hinder the systematic psychometric characterization of LLM outputs in assessment contexts.

Against this background, the present study applies a psychometric reliability framework to evaluate multimodal LLM performance on the complete 2021 TUS Second Administration. By integrating a broad landscape assessment with a structured multi-run reliability design and performing paired statistical comparisons across systems, this work establishes a reproducible methodological reference for characterizing AI-based assessment outcomes. The findings contribute to ongoing efforts in the behavioral sciences to understand how LLMs perform on structured assessment tasks designed to assess clinical knowledge and reasoning in human examinees, to evaluate their measurement properties, and to inform the responsible integration of AI into assessment research and practice.

## 2. Materials and Methods

### 2.1. Study Design

This study employed a cross-sectional dual-setup design, referring to a point-in-time benchmarking evaluation without longitudinal measurements, to comprehensively assess contemporary multimodal LLMs within a structured assessment framework. Data collection was conducted in October 2025. The dual-setup structure enabled both a broad comparative performance overview and a focused measurement stability analysis.

Setup 1 consisted of a single-run evaluation of ten proprietary and open-source models across all 240 items of the 2021 TUS Second Administration. This exploratory phase provided a comparative assessment of the current LLM ecosystem under uniform zero-shot conditions, meaning that models received each item without prior examples, demonstrations, or task-specific conditioning that could influence their responses (Zaghir et al., 2024).

Setup 2 implemented a multi-run assessment of six selected models representing diverse architectures and training paradigms. Each model completed five independent evaluation runs on the identical 240-item examination under the same standardized zero-shot conditions, enabling quantification of inter-run consistency, estimation of uncertainty around accuracy, and formal paired comparisons through McNemar testing (McNemar, 1947).

Across both setups, original TUS items, including all text and image material, were administered without modification. Models were accessed exclusively through publicly available web interfaces under standardized zero-shot prompting to reflect typical user-facing conditions and enable fair comparison across heterogeneous systems. All evaluation runs followed a standardized collection protocol to ensure methodological consistency and reproducibility.

Where models accepted direct image input, original examination images were provided alongside the corresponding items. For models without image-input capability, only item text and any accompanying image captions were used. Because only four image-based items were present in the TUS, the multimodal component was designed as an exploratory, hypothesis-generating submodule rather than a fully powered comparative analysis.

### 2.2. Assessment Instrument

The assessment instrument comprised the complete set of 240 multiple-choice items from the TUS 2021 Second Administration (5 September 2021). Each item presented five response options (A through E), requiring selection of the single best answer. The examination consists of two subtests: the Temel Tıp Bilimleri Testi (TTBT; items 1 through 120, covering basic medical sciences) and the Klinik Tıp Bilimleri Testi (KTBT; items 121 through 240, covering clinical medical sciences).

Four items (1.7%) included medical images such as radiographs, polarized-light microscopy, or magnetic resonance imaging. The remaining 236 items (98.3%) were text-only. All items were administered in their original Turkish wording to preserve authentic linguistic and cognitive complexity. Materials were obtained from the official ÖSYM repository. In accordance with copyright regulations, original items and answer keys are not reproduced; only aggregated performance data are reported (ÖSYM, 2021a, 2021b, 2021c; TUSData, n.d.).

The TUS uses ranking-based scoring without a fixed pass threshold, with specialty placement determined by relative performance among a cohort that may exceed 20,000 candidates. According to the official ÖSYM evaluation report for the 2021 administration, the highest observed scores were 115 out of 120 (95.83%) for TTBT and 111 out of 120 (92.50%) for KTBT. Because ÖSYM does not release individual-level data, it is unknown whether both maxima were achieved by a single candidate. A combined total of 226 out of 240 (94.17%) therefore represents a theoretical upper bound rather than verified single-candidate performance, although the value is used in this study only as an illustrative section-level reference, not as an individual benchmark (ÖSYM, 2021a, 2021b, 2021c; TUSData, n.d.).

### 2.3. Models Evaluated

Ten LLMs were selected to represent a balanced range of proprietary and open-source architectures, geographic origins, and parameter scales.

Proprietary models (*n* = 5) included GPT-5 (OpenAI, San Francisco, CA, USA), Claude Opus 4.1 (Anthropic, San Francisco, CA, USA), Gemini 2.5 Pro (Google LLC, Mountain View, CA, USA), Grok-4 (xAI, San Francisco, CA, USA), and ERNIE 4.5 Turbo (Baidu, Beijing, China).

Open-source models (*n* = 5) included DeepSeek V3.2 (DeepSeek, Hangzhou, China), Qwen3-Max (Alibaba Cloud, Hangzhou, China), Mistral Medium 3.1 (Mistral AI, Paris, France), Llama 3.3 70B Instruct (Meta AI, CA, USA), and Falcon-H1-34B-Instruct (Technology Innovation Institute, Abu Dhabi, United Arab Emirates).

Although Qwen3-Max and Mistral Medium 3.1 are proprietary hosted models, both originate from developers with established traditions of releasing open-source or open-weight model families that are widely adopted in academic research. To preserve group balance and acknowledge this architectural lineage, these models were classified within the open-source category. This categorization reflects the developers’ broader open-source orientation and the continuity of their respective model ecosystems rather than strict access modality. Setup 1 evaluated all ten models using a single-run protocol to characterize the broader LLM assessment landscape. Setup 2 conducted a focused multi-run reliability assessment of six models selected based on Setup 1 performance and architectural diversity: GPT-5 Pro (OpenAI), Claude Opus 4.1, Gemini 2.5 Pro, DeepSeek V3.2, Qwen3-Max, and Mistral Medium 3.1. The GPT-5 Pro variant was deliberately employed in Setup 2 rather than the standard GPT-5 used in Setup 1, because the multi-run reliability phase was intended to characterize the most capable available variant of each provider. Therefore, direct cross-setup numerical comparison between GPT-5 and GPT-5 Pro is not appropriate, and Setup 2 results for this provider should be interpreted as a separate model variant rather than a replication of Setup 1.

All models were accessed via public web interfaces.

### 2.4. Prompting Strategy

All models were evaluated using a standardized zero-shot prompting approach, meaning that no example items or solved samples were provided prior to evaluation. This approach ensured methodological comparability and prevented in-context learning effects.

Prompt construction followed the CO-STAR framework, which specifies Context, Objective, Style, Tone, Audience, and Response format (Kuerbanjiang et al., 2025; Sivarajkumar et al., 2024). The model was framed as an expert physician (Context), tasked with answering TUS multiple-choice items (Objective), using concise and clinically neutral language (Style and Tone), directed toward clinicians and trainees (Audience), and instructed to produce a table with item number and single-letter answer only (Response format). Explanations or additional text were prohibited to enable clean automated scoring. All items were presented in their original Turkish wording, with instructions provided in English, consistent with established multilingual LLM evaluation practices. The complete prompt is provided as Supplementary Material S1.

### 2.5. Data Collection

In Setup 1, each of the ten models completed a single evaluation run in a fresh browser session. All 240 items were presented sequentially. Outputs were checked for adherence to the required single-letter response format, and runs were restarted if deviations occurred.

In Setup 2, the same procedure was repeated five times per selected model, yielding 30 independent runs. Independence was ensured by using fresh browser sessions, clearing cache, and implementing temporal separation between runs. Item order followed the official TUS sequence across all runs.

### 2.6. Methodological Constraints

All evaluations were conducted through publicly accessible web chat interfaces rather than application programming interfaces (APIs). This approach does not allow control over sampling parameters such as temperature, top-p, or random seeds, and it provides no transparency regarding the precise model version active at the time of testing. Providers may update hosted models without notification, which limits reproducibility at the token level.

These constraints are inherent to web-based evaluation but reflect how most clinicians, students, and educators interact with LLMs in real-world assessment contexts.

### 2.7. Statistical Analysis

Accuracy in Setup 1 was defined as the proportion of correct responses out of 240 items. Each item was scored on the model’s single response in the structured answer table. The CO-STAR prompt produced consistently well-formed single-letter responses (A through E) across all administrations, requiring no rescoring or post-processing. Two-sided 95% confidence intervals were calculated using the Wilson score method (Newcombe, 1998; Brown et al., 2001).

For Setup 2, inter-run reliability was quantified using Cohen’s kappa coefficient (Cohen, 1960) across the full categorical response set (A through E). All 10 pairwise combinations of the five runs were evaluated for each model, and mean kappa with standard deviation summarized inter-run consistency. Interpretation followed the criteria of Landis and Koch (1977). Mean accuracy across the five runs (pooled across 1200 observations: 240 items multiplied by 5 runs) was reported with 95% Wilson score confidence intervals.

Per-run accuracy and Cohen’s kappa were computed on each model’s single response per run, with no aggregation or voting across runs. For the between-model paired comparisons only, a per-item majority-vote consensus across the five runs was generated for each model to obtain a single stable response vector per system; this consensus vector was used exclusively as the input for the pairwise McNemar tests and not for the accuracy or reliability estimates. Differences in accuracy between models were tested using McNemar’s exact test (McNemar, 1947). Chi-square statistics with continuity correction were computed alongside exact two-sided binomial *p*-values. Odds ratios (ORs) were calculated as the ratio of discordant pairs (b/c), with 95% confidence intervals computed using the log-Woolf method. Each model comparison comprised 240 paired observations. Two-sided *p*-values were adjusted for multiple comparisons using a Bonferroni correction across 15 pairwise tests (Sedgwick, 2014; Armstrong, 2014; Nakagawa, 2004).

Subgroup analyses compared image-containing items with text-only items. Differences in accuracy were examined using chi-square tests applied to aggregated contingency tables. Effect size was expressed as the absolute percentage-point difference, with 95% Wilson score confidence intervals.

Statistical analyses were conducted using Python 3.12 with standard statistical and data visualization libraries.

## 3. Results

This dual-setup evaluation assessed multimodal LLM measurement properties on the TUS. Setup 1 surveyed ten models in a single full exam run to characterize the assessment landscape and to inform selection for the reliability phase. Setup 2 performed a multi-run reliability assessment of six selected models (five independent runs each) to estimate central tendency, reproducibility, and paired differences.

### 3.1. Overall Performance Across Both Setups

Across both setups, contemporary multimodal LLMs achieved high absolute accuracy and demonstrated strong run-to-run agreement. Setup 1 provides a descriptive single-run landscape, whereas Setup 2 quantifies reproducibility through repeated evaluation. In Setup 1, three proprietary models exceeded 90% accuracy; in Setup 2, all six evaluated models demonstrated tightly clustered mean accuracies with minimal run-to-run variance (see Table 1).

#### 3.1.1. Setup 1: Assessment Landscape Survey

The single-run Setup 1 revealed a performance span from 72.08% to 92.92%. Top performers among the ten evaluated systems included Gemini 2.5 Pro (92.92%; 95% CI 89.2–95.5), Claude Opus 4.1 (90.83%; 95% CI 86.5–94.0), and GPT-5 (90.42%; 95% CI 86.3–93.6). Several open-source systems produced competitive results (Qwen3-Max 88.33%; Llama 3.3 70B 85.00%; Mistral Medium 3.1 84.58%), while other models scored lower (Grok-4 83.33%; DeepSeek V3.2 78.33%; ERNIE 4.5 Turbo 76.25%; Falcon-H1-34B 72.08%; 95% CI 66.0–77.6). The observed distribution guided the selection of six systems for the multi-run reliability assessment, ensuring representation of both high performers and architectural diversity (see Table 1).

#### 3.1.2. Setup 2: Multi-Run Performance and Measurement Stability

**Figure 1:**
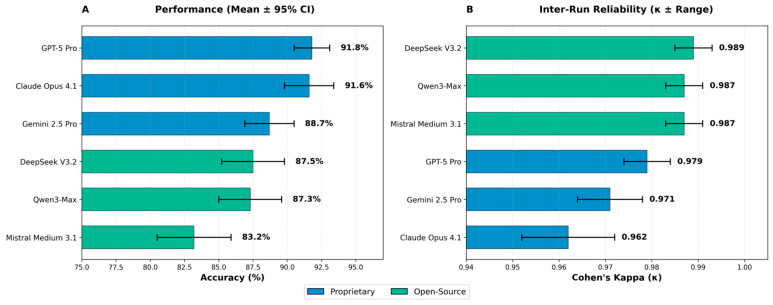
Setup 2 Performance (**A**) and Inter-Run Reliability (**B**). CI = confidence interval (Wilson score method); κ = Cohen’s kappa (categorical agreement on answer choices A through E). All models show ‘Almost perfect’ agreement (κ = 0.81–1.00) according to the Landis and Koch (1977) interpretation scale.

Setup 2 evaluated six LLMs across five fully independent runs. Inter-run agreement was almost perfect for all models, with high kappa values and narrow variability ranges (see Figure 1). Mean accuracies across runs reproduced the broad performance tiers observed in Setup 1, although exact ranking shifted slightly. Notably, the standard variant of one proprietary system ranked third in Setup 1 (see Table 1), while its enhanced Pro variant achieved the top position in the multi-run Setup 2, illustrating the impact of variant selection in the reliability phase (see Table 2 and Figure 1). Cross-setup correspondence between single-run and multi-run outcomes was moderate and consistent with expected stochastic variability in web-interface evaluations.

### 3.2. Pairwise Model Comparisons

**Figure 2:**
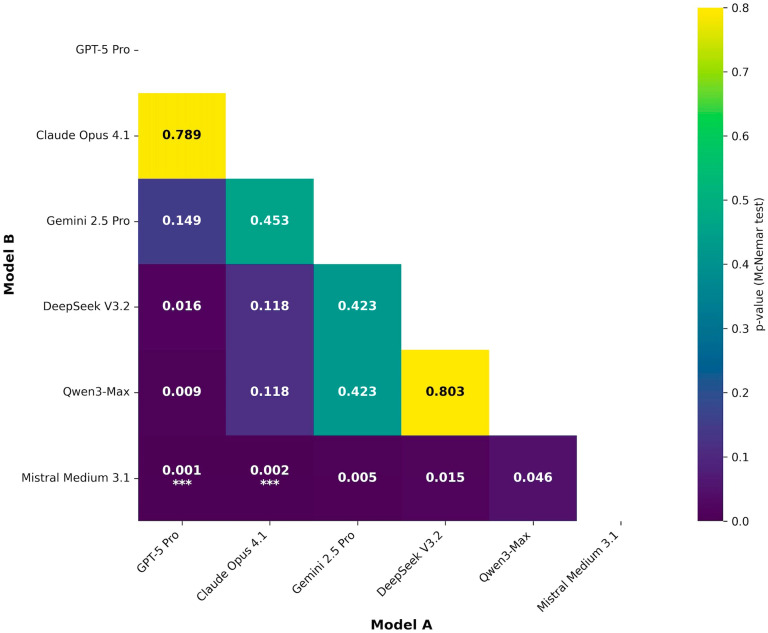
Pairwise statistical comparisons of model performance (Setup 2). *p*-values are from McNemar’s exact test based on 240 paired observations for each model pair. Familywise error was controlled using Bonferroni correction, providing a corrected significance threshold of α = 0.0033 (0.05/15 comparisons). Cells annotated with *** indicate contrasts that meet this corrected threshold. Color intensity reflects *p*-value magnitude.

Pairwise significance testing was conducted using McNemar’s exact test on 240 paired observations per comparison and corrected for multiple testing using Bonferroni adjustment (adjusted α = 0.0033). Only two contrasts met the corrected significance threshold. The leading proprietary system outperformed the lowest-ranked model with *p* < 0.001 and OR = 11.50 (95% CI 2.71–48.78). The second-ranked proprietary system also outperformed the lowest-ranked model with *p* = 0.002 and OR = 3.71 (95% CI 1.61–8.56). All other pairwise comparisons were not significant after adjustment. Full pairwise statistics, including exact *p*-values, test statistics, and odds ratios for all 15 contrasts, are provided in Table 3 and visualized in Figure 2.

### 3.3. Multimodal Performance: Image-Based Versus Text-Based Items

A subset of multimodal systems capable of visual interpretation was included in the exploratory image submodule. Because the TUS contains only four distinct image items, the image component was conceived as an illustrative rather than fully powered analysis of multimodal assessment capability.

In Setup 1, these four image items were administered to a small exploratory panel of models with image-processing functionality. Setup 2 subsequently included the same multimodal models and added two further open-source systems to create a balanced panel. Each model completed five independent runs, yielding 20 image attempts per model (4 items multiplied by 5 runs). Although multi-run aggregation increased the stability of point estimates, the number of distinct image items remained four, which inherently limits statistical power for image-text comparisons.

**Figure 3:**
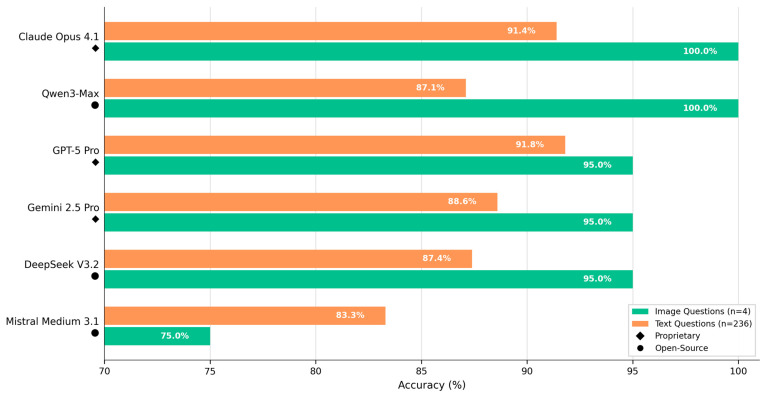
Image versus text item performance (Setup 2: multi-run multimodal panel). Green bars = image-item accuracy (*n* = 4 image items × 5 runs = 20 image attempts per model). Orange bars = text-item accuracy (*n* = 236 text items × 5 runs = 1180 text attempts per model). Diamond marker = proprietary model. Circle marker = open-source model. No image-versus-text comparison reached statistical significance in Setup 2.

Across the included models, accuracy on image-containing items was descriptively comparable to or slightly higher than accuracy on text-only items. Several models achieved perfect or near-perfect image scores while text accuracy showed broader variability. No image-text differences reached statistical significance in Setup 2 (all *p* > 0.05). For model-level visualization, see Figure 3, and for numerical details, see Table 4.

### 3.4. Cross-Setup Validation

Cross-setup validation compared single-run accuracy from Setup 1 with multi-run mean accuracy from Setup 2 for the six LLMs included in the multi-run cohort. Overall correspondence between the two setups was moderate. High-performing systems in Setup 1 generally remained within the upper performance tier in Setup 2, although individual ranking positions shifted. These shifts reflect both stochastic variability between runs and the deliberate use of an enhanced variant for one proprietary system in Setup 2, which introduced a systematic performance difference relative to Setup 1.

**Figure 4:**
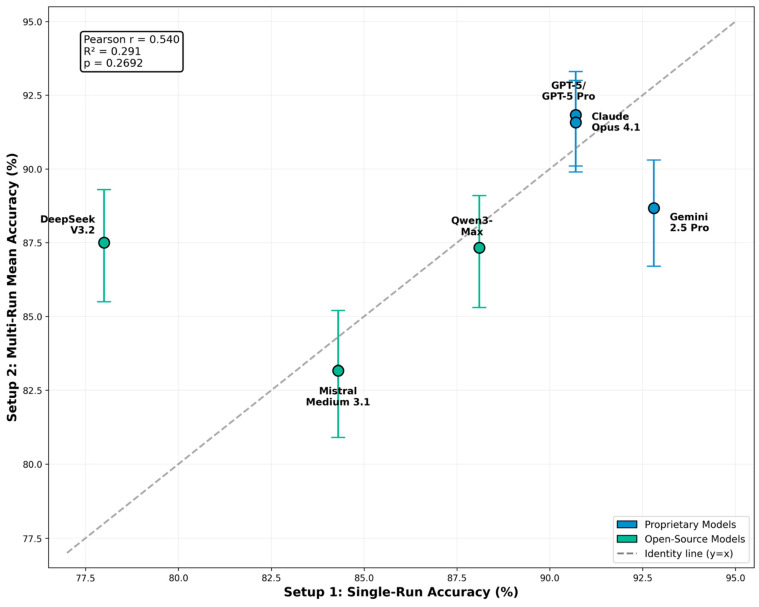
Cross-setup validation: single-run versus multi-run accuracy. Points represent per-model accuracy, with Setup 1 single-run results on the *x*-axis and Setup 2 five-run mean accuracy on the *y*-axis. Error bars show 95% confidence intervals for Setup 2 (Wilson score method). Dashed diagonal line indicates perfect agreement between setups.

Despite these variations, no system exhibited a discordant pattern suggestive of measurement instability or regression across setups. The relationship between single-run and multi-run outcomes is visualized in Figure 4, with most systems clustering near the identity line. For a compact numeric comparison across setups, see Table 1 and Table 2.

### 3.5. AI Performance Relative to Human Assessment Benchmarks

To contextualize LLM performance, accuracy was compared against the highest human subtest scores reported by ÖSYM for the 2021 TUS Second Administration. These maxima comprise 115 correct answers for the Basic Medical Sciences Test (TTBT) and 111 for the Clinical Medical Sciences Test (KTBT), yielding a combined section-maximum reference of 226 out of 240 (94.17%).

**Figure 5:**
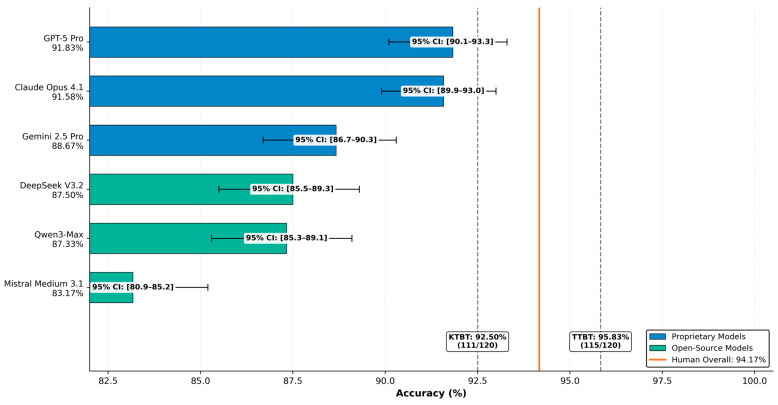
Setup 2 multi-run LLM accuracy values plotted alongside the section-maximum scores reported by ÖSYM for the 2021 TUS Second Administration. The reference lines (TTBT 95.83%; KTBT 92.50%) and the combined value of 226/240 (94.17%) reflect aggregate section maxima only; they are not linked to individual candidates and do not represent observed individual human performance. The figure is illustrative and must not be interpreted as evidence of parity, equivalence, or comparable underlying response processes between LLMs and human examinees. CI = confidence interval (Wilson score method); TTBT = Basic Medical Sciences Test; KTBT = Clinical Medical Sciences Test.

This reference value is presented for illustrative orientation only. Because ÖSYM does not release individual-level score data, the combined section maxima cannot be attributed to any single examinee, and the value does not represent observed individual human performance. Comparisons between leading LLMs and this reference value should therefore not be interpreted as evidence of parity or equivalence with human examinees. They serve solely to anchor absolute LLM accuracy values within the score range observed at section level for the 2021 TUS Second Administration (see Figure 5).

## 4. Discussion

This study provides a comprehensive assessment-focused evaluation of contemporary multimodal LLMs on the complete TUS, integrating a broad single-run landscape survey with a multi-run reliability design grounded in established psychometric methodology. In contrast to prior non-English evaluations, many of which restricted analysis to partial item sets or excluded visual content owing to earlier model limitations (Jung et al., 2023), this benchmark uses every original item and incorporates both text-based and image-based content. Critically, the present work moves beyond reporting performance metrics toward examining what these metrics imply for assessment science and measurement theory. Throughout the following discussion, performance and reliability are interpreted strictly at the level of observable accuracy and inter-run agreement. The design measures whether LLMs select correct response options and whether they do so consistently across repeated administrations; it does not measure whether the underlying response process resembles human clinical reasoning, and statements about cognitive equivalence are deliberately avoided.

### Three Overarching Findings Emerge

The first finding concerns accuracy and its interpretation within a structured assessment context. Several systems achieved high accuracy on the TUS despite the cross-lingual demands of a Turkish-language examination and the curricular distinctiveness of the underlying medical training system. The strongest proprietary systems converged near an accuracy ceiling of approximately 90% (Table 1 and Table 2), consistent with observations from other national licensing examinations (Kung et al., 2023; Knoedler et al., 2024; Nori et al., 2023; Roos et al., 2023; Mihalache et al., 2023; Masters, 2019; Penny et al., 2024; Gilson et al., 2023; Abbas et al., 2024; Bicknell et al., 2024; Mihalache et al., 2024; Garabet et al., 2023; Balu et al., 2025; Yang & Chen, 2025). However, high accuracy alone does not establish that the cognitive constructs targeted by the examination are being engaged. When AI systems achieve scores numerically comparable to high-performing human examinees, identical accuracy values may arise from fundamentally different response mechanisms: clinical reasoning, knowledge integration and diagnostic judgment in human examinees, versus statistical associations learned from training-data distributions and pattern-based response selection in LLMs. Numerical similarity at the score level therefore does not justify any inference of process-level equivalence. This distinction is central to assessment science, where the validity of score interpretations depends not only on what is answered correctly but on the processes that produce the answer. The clustering of leading systems near a shared performance plateau further suggests that performance gains may be approaching a ceiling under the specific assessment conditions studied here, a pattern that may point toward diminishing returns of model scaling under conventional item formats. Item-level variability likely reflects differences in local guideline traditions and diagnostic frameworks embedded within Turkish medical education, and if models implicitly translate prompts into an English-language latent space, responses may reflect Anglophone medical norms rather than locally relevant instructional materials.

The second finding concerns measurement stability. All kappa values exceeded the “Almost perfect” threshold defined by Landis and Koch (1977), with minimal run-to-run variance (Figure 1B). This level of consistency positions LLMs as systems whose outputs can be characterized using the formal vocabulary of psychometrics, while remaining conceptually distinct from human examinees: high run-to-run agreement in LLMs reflects algorithmic determinism and constrained output variability under stable prompting conditions, rather than the within-person cognitive stability that underlies human test–retest reliability. However, high reliability is a necessary but not sufficient condition for valid measurement. Reliability without evidence of construct-relevant response processes remains psychometrically incomplete, and the observation that LLMs produce highly consistent outputs does not resolve whether correct responses reflect clinical reasoning or retrieval from memorized training content. Single-run evaluations remain suitable for broad landscape analysis, but multi-run designs provide added value for inferential comparisons and for contextualizing stability in hosted systems that may undergo undocumented updates. The moderate correspondence between single-run and multi-run results (Figure 4) underscores the importance of rigorous methodological design in LLM-based assessment studies.

The third finding is exploratory and concerns multimodal assessment capability. With only four distinct image items in the TUS, the multimodal component is hypothesis-generating rather than confirmatory, and the descriptive observation that several frontier systems achieved image-item accuracy comparable to or slightly exceeding their text-item accuracy must be interpreted with caution. The pattern is compatible with the hypothesis that medical imagery may no longer represent a categorical weakness for current multimodal systems, but firm conclusions cannot be drawn from this small subset. In addition, TUS image items are typically embedded in detailed clinical stems that may carry diagnostic information independent of the visual content, so any equivalence between image- and text-item performance may partly reflect text-stem redundancy rather than genuine visual reasoning. Dedicated multimodal item banks with image-only stems are required to delineate the boundaries of current visual reasoning capacity (Figure 3; Table 4).

Beyond the three core findings, the present results raise questions about construct validity. If items that differentiate among human examinees at varying competence levels fail to differentiate between systems relying on fundamentally different response strategies, then current item formats may lack the discriminative capacity needed to distinguish reasoning-based from retrieval-based performance. Future research should examine item-level difficulty concordance between human and AI respondents: concordant difficulty profiles would suggest that the assessment captures construct-relevant variance that generalizes across respondent types, whereas divergent profiles would indicate that certain items are differentially susceptible to pattern-based solving and may warrant revision. This distinction is central not only to assessment science but also to the behavioral sciences more broadly, where understanding the relationship between observable responses and underlying cognitive processes is fundamental. Convergence among leading systems may also extend to systematic errors. Items on which multiple top-performing models select the same incorrect option may indicate either flawed items, ambiguous wording, or shared biases in training data common across providers. A formal item-level error-convergence analysis combined with item-response-theory modelling would directly test these hypotheses and is a promising direction for future work building on the present multi-run dataset.

From a behavioral-science perspective, examinee performance on high-stakes multiple-choice assessments arises from the interaction of recognition, retrieval, and analytic decision processes. The current evaluation captures only the output side of this process and cannot distinguish whether AI responses reflect retrieval-dominant pattern matching, analytic problem-solving over textual cues, or a mixture thereof. This distinction is conceptually important for interpreting any apparent performance parity between AI systems and human candidates and motivates future work incorporating reasoning-trace analysis and process-tracing methods.

The results also carry implications for fairness in AI-augmented assessment. LLMs are trained predominantly on English-language corpora, creating a structural asymmetry when applied to non-English assessments. Performance differences across systems on the Turkish-language TUS suggest that language-specific training exposure may introduce differential functioning independent of the cognitive demands of the assessment. In contexts where AI tools support item development, scoring, or feedback, such differences could propagate systematic biases. The opacity of training data further raises concerns about content contamination: because the 2021 TUS item set is publicly accessible, leakage cannot be excluded, and elevated accuracy may partially reflect memorization. Future benchmarking should incorporate explicit leakage checks, for example, by testing on held-out or synthetic item variants or by reporting vendor-provided training cutoff dates.

From a practical standpoint, systems demonstrating high accuracy and stability may be explored for supervised integration into assessment-related workflows, including item pre-screening, formative feedback generation, and quality assurance of examination content. Because leading systems cluster near a shared accuracy plateau, practical considerations such as accessibility, cost, and integration capability may become increasingly relevant for selection decisions.

Additional limitations include the small number of image items; reliance on publicly accessible web interfaces, which precludes control over sampling parameters such as temperature and top-p, prevents version locking, and exposes the evaluation to undocumented provider-side updates such as silent model refreshes or vendor-side A/B routing that cannot be excluded by any user-side mechanism, with the October 2025 data collection window therefore functioning as the effective version anchor and all reported results to be interpreted as the performance of the listed provider labels accessed via their respective public web interfaces during that window rather than the performance of fixed, locally reproducible model checkpoints; the use of GPT-5 in Setup 1 and GPT-5 Pro in Setup 2, which limits direct cross-setup comparability for this provider; zero-shot prompting that does not capture the upper bound of system capability; restriction to a single examination cycle; the use of five repeated runs per model in Setup 2, which yields high mean inter-run agreement but provides only limited resolution for the variance components that a more granular reliability decomposition would require; the omission of item response theory (IRT) and other item-level psychometric models, given that ÖSYM does not release the item-level human response data needed for joint calibration and the present design targets system-level rather than item-level characterization; the conceptual distinction between the computational reproducibility of LLM outputs under stable prompting conditions reported here and the within-person psychometric reliability observed in human test–retest designs, which should not be equated; and the absence of analyses targeting calibration, uncertainty quantification, and hallucination risk, which fall outside an accuracy-and-agreement-based evaluation framework and warrant dedicated study. Two further limitations warrant explicit emphasis. First, item-level human performance data for the 2021 TUS are not publicly released by ÖSYM, so comparisons between LLMs and human examinees can only be made at aggregate section-score level. Second, this study evaluates response correctness only; reasoning traces, error typology, and process-level alignment with human decision-making were not analyzed and cannot be inferred from accuracy. Future studies incorporating reasoning-trace analyses and item-level error-pattern alignment would directly address this latter constraint, complementing the leakage-mitigation strategies noted above.

Despite these constraints, the TUS provides a valuable multilingual and multimodal assessment benchmark owing to its broad domain coverage, cross-lingual demands, and standardized scoring.

## 5. Conclusions

This study demonstrates that contemporary multimodal LLMs achieve high and reproducible accuracy on a national medical licensing examination administered in Turkish. Inter-run agreement exceeded 0.96 across all models, indicating high run-to-run reproducibility under realistic conditions. Comparisons against an illustrative section-maximum reference are descriptive only and do not support claims of parity with human examinees.

Beyond performance, the findings contribute to AI-based assessment research in three ways. First, the multi-run reliability framework provides a transferable approach for characterizing the psychometric properties of LLM outputs, addressing the current reliance on single-run accuracy reports. Second, the convergence of top-performing systems near a shared performance ceiling raises questions about the discriminative capacity of existing item formats when applied to both human and AI respondents, with implications for construct validity. Third, cross-lingual performance differences highlight the importance of fairness and representational equity in AI-supported assessment contexts.

LLMs exhibit highly reproducible response patterns across repeated administrations, positioning them as objects of AI-based assessment research at the level of measurable outcomes. This reproducibility should not be interpreted as evidence of human-equivalent cognitive processing; high reliability does not establish that equivalent reasoning processes are engaged, and the distinction between reasoning-based and pattern-based performance remains unresolved.

These findings suggest that the integration of AI into assessment contexts warrants continued investigation into whether existing assessment formats adequately differentiate between reasoning-based and pattern-based response strategies, particularly in high-stakes examinations.

## Figures and Tables

**Table 1:** Model Performance Summary: Setup 1 (Single Run) and Setup 2 (Multi-Run).

Model	Setup	Type	Accuracy (%)	95% CI	SD (%)	κ Mean
Setup 1 (*n* = 10 models, single run)						
Gemini 2.5 Pro	1	Proprietary	92.92	89.2–95.5	-	-
Claude Opus 4.1	1	Proprietary	90.83	86.5–94.0	-	-
GPT-5	1	Proprietary	90.42	86.3–93.6	-	-
Qwen3-Max	1	Open-Source	88.33	83.7–91.9	-	-
Llama 3.3 70B	1	Open-Source	85.00	79.9–89.2	-	-
Mistral Medium 3.1	1	Open-Source	84.58	79.4–88.9	-	-
Grok-4	1	Proprietary	83.33	78.0–87.8	-	-
DeepSeek V3.2	1	Open-Source	78.33	72.6–83.3	-	-
ERNIE 4.5 Turbo	1	Proprietary	76.25	70.4–81.4	-	-
Falcon-H1-34B	1	Open-Source	72.08	66.0–77.6	-	-
Setup 2 (*n* = 6 models, 5 runs each)						
GPT-5 Pro	2	Proprietary	91.83	90.1–93.3	0.33	0.979
Claude Opus 4.1	2	Proprietary	91.58	89.9–93.0	1.30	0.962
Gemini 2.5 Pro	2	Proprietary	88.67	86.7–90.3	0.55	0.971
DeepSeek V3.2	2	Open-Source	87.50	85.5–89.3	0.27	0.989
Qwen3-Max	2	Open-Source	87.33	85.3–89.1	0.34	0.987
Mistral Medium 3.1	2	Open-Source	83.17	80.9–85.2	0.33	0.987

CI = Confidence Interval (Setup 1 and Setup 2: Wilson score method); SD = Standard Deviation; κ = Cohen’s kappa (inter-run agreement).

**Table 2:** Multi-Run Performance Summary and Inter-Run Reliability (Setup 2).

Rank	Model	Type	Run 1 (%)	Run 2 (%)	Run 3 (%)	Run 4 (%)	Run 5 (%)	Mean (%)	SD (%)	95% CI	κ Mean	κ Range (Min–Max)	Performance Tier
1	GPT-5 Pro	Proprietary	91.67	92.08	92.08	91.25	92.08	91.83	0.33	90.1–93.3%	0.979	0.969–0.990	Tier 1 (≥90%)
2	Claude Opus 4.1	Proprietary	90.83	90	90.83	92.92	93.33	91.58	1.3	89.9–93.0%	0.962	0.943–0.990	Tier 1 (≥90%)
3	Gemini 2.5 Pro	Proprietary	88.75	88.33	89.58	88.75	87.92	88.67	0.55	86.7–90.3%	0.971	0.958–0.979	Tier 2 (85–90%)
4	DeepSeek V3.2	Open-Source	87.5	87.92	87.5	87.5	87.08	87.5	0.27	85.5–89.3%	0.989	0.984–0.995	Tier 2 (85–90%)
5	Qwen3-Max	Open-Source	87.08	87.92	87.5	87.08	87.08	87.33	0.34	85.3–89.1%	0.987	0.979–1.000	Tier 2 (85–90%)
6	Mistral Medium 3.1	Open-Source	82.92	82.92	83.33	83.75	82.92	83.17	0.33	80.9–85.2%	0.987	0.969–1.000	Tier 3 (<85%)

SD = standard deviation; CI = confidence interval (Wilson score method); κ = Cohen’s kappa (inter-run categorical agreement). All models show ‘Almost perfect’ agreement (κ = 0.81–1.00) according to the Landis and Koch (1977) interpretation scale.

**Table 3:** Pairwise Model Comparisons (Setup 2): McNemar’s Test with Bonferroni Correction.

Comparison	Both Correct	A✓ B✗	A✗ B✓	χ^2^	*p*-Value	Odds Ratio	95% CI (OR)	Sig.
GPT-5 Pro vs. Claude Opus 4.1	212	8	6	0.07	0.789	1.33	0.46–3.84	
GPT-5 Pro vs. Gemini 2.5 Pro	211	9	3	2.08	0.149	3.00	0.81–11.08	
GPT-5 Pro vs. DeepSeek V3.2	208	12	2	5.79	0.016	6.00	1.34–26.81	
GPT-5 Pro vs. Qwen3-Max	209	11	1	6.75	0.009	11.00	1.42–85.20	
GPT-5 Pro vs. Mistral Medium 3.1	197	23	2	16.00	<0.001	11.50	2.71–48.78	✓
Claude Opus 4.1 vs. Gemini 2.5 Pro	208	10	6	0.56	0.453	1.67	0.61–4.59	
Claude Opus 4.1 vs. DeepSeek V3.2	204	14	6	2.45	0.118	2.33	0.90–6.07	
Claude Opus 4.1 vs. Qwen3-Max	204	14	6	2.45	0.118	2.33	0.90–6.07	
Claude Opus 4.1 vs. Mistral Medium 3.1	192	26	7	9.82	0.002	3.71	1.61–8.56	✓
Gemini 2.5 Pro vs. DeepSeek V3.2	205	9	5	0.64	0.423	1.80	0.60–5.37	
Gemini 2.5 Pro vs. Qwen3-Max	205	9	5	0.64	0.423	1.80	0.60–5.37	
Gemini 2.5 Pro vs. Mistral Medium 3.1	194	20	5	7.84	0.005	4.00	1.50–10.66	
DeepSeek V3.2 vs. Qwen3-Max	202	8	8	0.06	0.803	1.00	0.38–2.66	
DeepSeek V3.2 vs. Mistral Medium 3.1	196	14	3	5.88	0.015	4.67	1.34–16.24	
Qwen3-Max vs. Mistral Medium 3.1	192	18	7	4.00	0.046	2.57	1.07–6.16	

Significant at Bonferroni-corrected α = 0.0033 (α = 0.05/15 comparisons). McNemar χ^2^ (continuity corrected) and two-sided exact *p*-values are reported. OR = Odds Ratio, representing the odds that Model A is correct when the two models disagree. 95% confidence intervals for odds ratios were computed using the log-Woolf (asymptotic) method. A✓ B✗ = Model A correct, Model B incorrect. A✗ B✓ = Model A incorrect, Model B correct. ✓ = comparison significant at Bonferroni-corrected threshold (α = 0.0033).

**Table 4:** Image Versus Text Performance Across Both Setups.

Model	Setup	Image Accuracy (%)	Image Correct/Total	Text Accuracy (%)	Text Correct/ Total	Difference (pp)	χ^2^	Sig.
Setup 1 (4 image questions per model, single run)								
Gemini 2.5 Pro	1	100	4/4	92.8	219/236	+7.20	—	—
Claude Opus 4.1	1	100	4/4	90.7	214/236	+9.30	—	—
GPT-5	1	75	3/4	90.7	214/236	−15.70	—	—
Qwen3-Max	1	100	4/4	88.1	208/236	+11.90	—	—
Setup 2 (20 image attempts per model: 4 questions × 5 runs)								
GPT-5 Pro	2	95	19/20	91.78	1083/1180	+3.22	0.27	No
Claude Opus 4.1	2	100	20/20	91.44	1079/1180	+8.56	1.87	No
Gemini 2.5 Pro	2	95	19/20	88.56	1045/1180	+6.44	0.81	No
DeepSeek V3.2	2	95	19/20	87.37	1031/1180	+7.63	1.05	No
Qwen3-Max	2	100	20/20	87.12	1028/1180	+12.88	2.95	No
Mistral Medium 3.1	2	75	15/20	83.31	983/1180	−8.31	0.97	No

pp = percentage points. Difference = Image Accuracy minus Text Accuracy. Setup 1: single-run image attempts (4 image items per model). Setup 2: multi-run image attempts (4 items × 5 runs per model). χ^2^ = Chi-square test for image versus text performance difference. None of the Setup 2 comparisons reached statistical significance (α = 0.05).

## Data Availability

All examination items used in this study are publicly accessible via the official ÖSYM repository. The original question materials of the 2021 TUS Second Administration (TTBT and KTBT) can be found at the following sources: https://dokuman.osym.gov.tr/pdfdokuman/2021/TUSDONEM2/CS/tusdonem_2_TTBT.pdf (accessed on 30 March 2026), https://dokuman.osym.gov.tr/pdfdokuman/2021/TUSDONEM2/CS/tusdonem_2_KTBT.pdf (accessed on 30 March 2026).

## Supplementary Materials

The following supporting information can be downloaded at: https://www.mdpi.com/article/10.3390/bs16050822/s1. Supplementary Material S1: complete prompt.

## Abbreviations

The following abbreviations are used in this manuscript:

AI	Artificial Intelligence
API	Application Programming Interfaces
CI	Confidence Interval
CO-STAR	Context, Objective, Style, Tone, Audience, Response format
IRT	Item Response Theory
KTBT	Klinik Tıp Bilimleri Testi (Clinical Medical Sciences Test)
LLM	Large Language Model
MLLM	Multimodal Large Language Model
OR	Odds Ratio
ÖSYM	Ölçme, Seçme ve Yerleştirme Merkezi (Assessment, Selection and Placement Center)
SD	Standard Deviation
TTBT	Temel Tıp Bilimleri Testi (Basic Medical Sciences Test)
TUS	Tıpta Uzmanlık Eğitimi Giriş Sınavı (Turkish Medical Specialization Entrance Examination)
USMLE	United States Medical Licensing Examination
