# Supplementary material for: Artificial Intelligence in Medical Assessment: Reliability and Performance of Multimodal Large Language Models in a High-Stakes Licensing Examination
Source: Behav Sci (Basel). 2026 May 19;16(5):822. doi: 10.3390/bs16050822 (PMC13203780; doi:10.3390/bs16050822)
Supplement: Supplementary file 1 [file behavsci-16-00822-s001.zip › behavsci-4260489-supplementary.pdf]

Supplementary Material S1 complete prompt template.

Context: You are an expert physician.

Objective: Answer the Turkish Medical Specialization Entrance Examination (TUS) multiple-choice items provided below. Each item presents five answer options (A through E); exactly one option is correct.

Style: Concise.

Tone: Clinically neutral.

Audience: Clinicians and trainees.

Response format: A single table with two columns, "Question" and "Answer". For each item, place the question number in the first column and a single letter (A, B, C, D, or E) in the second column. Do not include explanations, justifications, reasoning, or any text outside the table.
